# In vivo diagnosis of TDP-43 proteinopathies: in search of biomarkers of clinical use

**DOI:** 10.1186/s40035-024-00419-8

**Published:** 2024-06-03

**Authors:** Juan I. López-Carbonero, Irene García-Toledo, Laura Fernández-Hernández, Pablo Bascuñana, María J. Gil-Moreno, Jordi A. Matías-Guiu, Silvia Corrochano

**Affiliations:** Neurological Disorders Group, Hospital Clínico San Carlos, Instituto de Investigación Sanitaria San Carlos (IdISSC), 28040 Madrid, Spain

**Keywords:** TDP-43 proteinopathy, Biomarkers, Early diagnosis, ALS, FTD, LATE

## Abstract

TDP-43 proteinopathies are a heterogeneous group of neurodegenerative disorders that share the presence of aberrant, misfolded and mislocalized deposits of the protein TDP-43, as in the case of amyotrophic lateral sclerosis and some, but not all, pathological variants of frontotemporal dementia. In recent years, many other diseases have been reported to have primary or secondary TDP-43 proteinopathy, such as Alzheimer’s disease, Huntington’s disease or the recently described limbic-predominant age-related TDP-43 encephalopathy, highlighting the need for new and accurate methods for the early detection of TDP-43 proteinopathy to help on the stratification of patients with overlapping clinical diagnosis. Currently, TDP-43 proteinopathy remains a post-mortem pathologic diagnosis. Although the main aim is to determine the pathologic TDP-43 proteinopathy in the central nervous system (CNS), the ubiquitous expression of TDP-43 in biofluids and cells outside the CNS facilitates the use of other accessible target tissues that might reflect the potential TDP-43 alterations in the brain. In this review, we describe the main developments in the early detection of TDP-43 proteinopathies, and their potential implications on diagnosis and future treatments.

## Background

TDP-43 proteinopathies consist of a group of neurodegenerative diseases defined by the pathological presence of misfolded proteins and insoluble deposits of the transactive response DNA-binding protein of 43 kDa (TDP-43) in the central nervous system (CNS), in association with progressive neuronal loss and gliosis [1]. Pathological TDP-43 dysfunction and aggregation is associated with devastating diseases such as amyotrophic lateral sclerosis (ALS) and frontotemporal dementia (FTD), which are responsible for a high socioeconomic and health burden [2, 3].

A crucial issue in neurodegenerative diseases, especially in TDP-43 proteinopathies, is the fact that the same clinical phenotype can be related to different proteinopathies, and at the same time TDP-43 proteinopathy can be found in other different clinical disorders [4]. Thus, there is a pressing need to develop objective biomarkers related to the pathophysiology of the disease, as a potentially useful tool to assist in the correct early clinical diagnosis and a starting point for upcoming therapies targeting TDP-43 pathology [5].

To date, the diagnostic confirmation of the underlying TDP-43 proteinopathy requires a histopathological post-mortem study of the brain or spinal cord. It is then essential that we can identify the underlying proteinopathy in the heterogeneous overlapping clinical neurological disorders at pre-symptomatic or early disease stages. The experience from the most abundant and extensively studied neurodegenerative disease, Alzheimer’s disease (AD), with specific protein-based neuroimaging positron emission tomography (PET) radiotracers and the successful measurement of amyloid beta species (Aβ42, Aβ40), tau and phosphorylated tau 181 (p-tau181) in cerebrospinal fluid (CSF), has paved the way to the development of anti-amyloid therapies and other disease-modifying therapies [6]. Those successful studies have inspired future research on accurate protein-based biomarkers and treatments for other proteinopathies.

In this review, we recapitulate most of the recent advances in search for in vivo detection of TDP-43 proteinopathies for clinical use, classifying the different approaches into four main categories: (1) detection of soluble TDP-43 in biofluids, (2) functional and structural neuroimaging directly or indirectly associated with TDP-43, (3) detection of aberrant TDP-43 in cells and tissues outside the CNS, and (4) indirect detection of TDP-43 loss-of-function splicing by cryptic exon neoepitopes.

## Review methods

We searched original articles, reviews, clinical reports, systematic reviews and meta-analyses available in PubMed-indexed journals by the date of January 9, 2024, using the following keywords: “TDP-43”, “TDP43”, “TARDBP”, “TDP-43” [AND] “proteinopathy”, “TDP-43” [AND] (“amyotrophic lateral sclerosis” [OR] “frontotemporal dementia” [OR] “frontotemporal lobar degeneration”), “TDP-43” [AND] “biomarkers”, “TDP-43” [AND] (“plasma” [OR] “blood” [OR] “serum”), “TDP-43” [AND] (CSF [OR] “cerebrospinal fluid”), “TDP-43” [AND] “positron emission tomography”,”TDP-43” [AND] (“MRI” [OR] “neuroimaging”), “TDP-43” [AND] (“muscle” [OR] “adipose tissue” [OR] “fat” [OR] “adipocyte” [OR] “liver”), “TDP-43″ [AND] (“skin” [OR] “fibroblasts”)”, “TDP-43″ [AND] (“lymphocytes” [OR] “lymphomonocytes” [OR] “PBMC”), and “TDP-43″ [AND] “ALS” [AND] “nerve”. Articles in English, Spanish and French were considered for review. A few articles of interest published in German, Japanese, Chinese and Russian were disregarded.

## Structure and function of TDP-43

TDP-43 was first characterized in 1995 as a novel protein binding to transactive response (TAR) DNA sequence motifs of human immunodeficiency virus type 1, with a critical role in the activation of viral gene expression [7]. In 2006, Neumann et al. found deposits of ubiquitinated and hyperphosphorylated TDP-43 aggregates in brain tissues from sporadic ALS and ubiquitin-positive, tau-negative frontotemporal lobar degeneration, previously known as U-FTLD [8]. This breakthrough marked the beginning of more than two decades of intensive research on TDP-43 structure, function and potential mechanisms of disease.

**Fig. 1:**
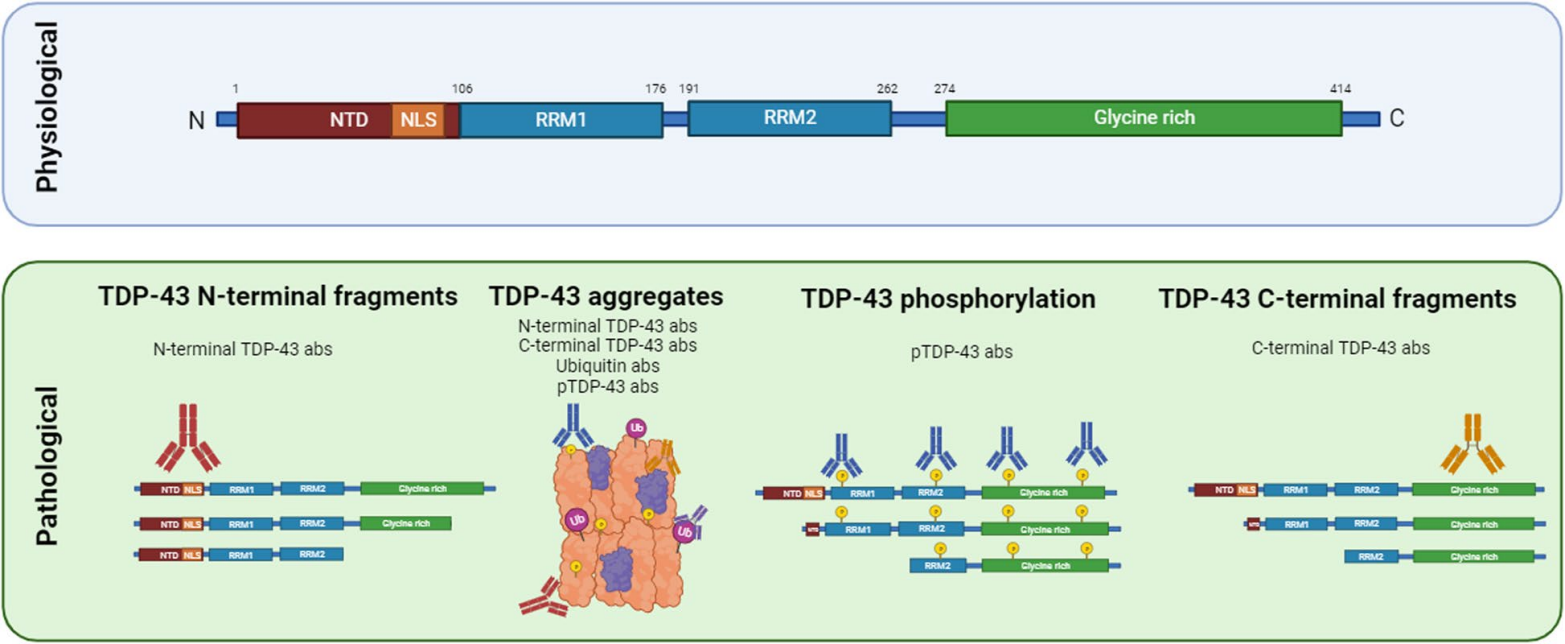
Physiological and pathological species of TDP-43. Upper: Schematic view of physiological structure of TDP-43, including the N-terminal domain (NTD), two RNA recognition motifs (RRM) and the glycine-rich C-terminal domain (CTD). Lower: Pathological species of TDP-43 and the antibodies that are mostly used for the detection of different fragments and aggregates, and the part of the protein they recognize

Encoded by the gene *TARDBP* (1p36.22), TDP-43 is an RNA/DNA-binding protein, classified within the heterogeneous nuclear ribonucleoprotein (hnRNP) family. The canonical TDP-43 is composed of 414 amino acids and has a structural organization characterized by a N-terminal domain (NTD, residues 1–102) including a nuclear localization sequence (NLS), two RNA-recognition motifs RRM1 (residues 106–177) and RRM2 (residues 192–259), and a C-terminal domain (CTD, residues 274–414), which is in turn subdivided into two glycine-rich regions and an amyloidotic core with a hydrophobic region (residues 318–340) and a prion-like glutamine-asparagine (Q/N)-rich region which are structurally prone to form amyloid-like fibrils [9]. TDP-43 was also thought to contain a nuclear export sequence within RRM2 (residues 239–250); however, most recent evidence supports that TDP-43 predominantly exits the nucleus by passive diffusion [10] (Fig. 1, upper panel).

TDP-43 participates in different cellular processes: regulation of RNA metabolism (RNA processing, cryptic splicing, RNA transport and microRNA biogenesis), stress response, protein quality control system, mitochondrial autophagy, vesicle transport and axonal transport [1]. Besides, TDP-43 controls the expression of synaptic proteins, such as synaptotagmin and synapsin I, and is present in postsynaptic dendrites, where it is involved in local protein translation [11].

## Physiological and pathological species of TDP-43

The predominant subcellular localization of TDP-43 in physiological condition is the nucleus. Upon different situations and insults, TDP-43 is translocated into the cytoplasm to develop a number of functions in, e.g., mRNA stability and transport, regulation of translation, processing of micro-RNA and stress response [12]. As a result, TDP-43 may be found in different cytoplasmic subcellular compartments, including endoplasmic reticulum, mitochondria, and liquid–liquid phase separation (LLPS) membraneless organelles, such as RNA granules and stress granules [13]. TDP-43 structure is prone to dimerization and oligomerization via its NTD [14]. This process of dimerization is required for splicing activity [15]. Eventually, when TDP-43 function is no longer required or cell stress has ended, the protein returns into the nucleus via importin α/β recognition of the NLS [16].

Under pathologic conditions, TDP-43 is observed permanently translocated to the cytoplasm, leading to nuclear depletion of TDP-43, causing impairment of its nuclear functions [17]. Loss of physiological dimerization of TDP-43 has resulted in a critical determinant for TDP-43 oligomerization and aggregation [18]. As a matter of fact, mutations in *TARDBP* affecting the NLS, such as A90V, prevent the nuclear import of TDP-43, leading to cytoplasmic sequestering and aggregation [14].

In addition, caspase-mediated cleaved species of the protein of ~ 35 and ~ 25 kDa have been observed in brain tissues of ALS and FTD-TDP patients [19]. These C-terminal fragments (CTFs) lack their NLS and become sequestered in the cytoplasm, where they expose their prion-like amyloidogenic regions, leading to protein aggregation and cytotoxicity [20]. Of note, CTFs are characteristic in brain pathology, but are rarely detected in the spinal cord, where TDP-43 deposits comprise the full-length protein, suggesting CTFs may not be a prerequisite for neurodegeneration [21]. Oligomers and amyloid-like fibrils of TDP-43 pathological species are deposited in the cytoplasm and in neurites (Fig. 1, lower panel). TDP-43 undergoes post-translational modifications as well, of which the most studied are phosphorylation and ubiquitination. Constant sites for aberrant phosphorylation on full-length TDP-43 and CTFs are located in the CTD (serines 379, 403/404 and 409/410) and their detection is considered a hallmark of disease in TDP-43 proteinopathies [10]. Therefore, pathological criteria for TDP-43 proteinopathy include the presence of intracytoplasmic inclusions of native, cleaved and phosphorylated TDP-43 species, in association with ubiquitin and p62 [22]. Both phosphorylation and ubiquitination are considered late phenomena that represent an attempt by the cellular machinery to evade protein aggregation and cytotoxicity [9].

## TDP-43 proteinopathies and clinically related disorders

A number of diseases involve TDP-43 as their primary neuropathology, in which a relevant pathogenic role has been postulated [4]. Main diseases related to TDP-43 pathology comprise most sporadic forms of ALS and about 50% of FTD (FTD-TDP), especially behavioral variant and semantic primary progressive aphasia [23, 24] as well as cognitive impairment associated with limbic-predominant age-related TDP-43 encephalopathy [25] and Perry syndrome [26]. TDP-43 proteinopathy in skeletal muscle cells is a common finding in sporadic inclusion body myositis (IBM) [27]. Facial onset motor and sensitive neuropathy (FOSMN) has also been associated with TDP-43 proteinopathy, and is currently considered a clinical variant of ALS [28]. Most genetic forms of FTD and ALS are also related to TDP-43 proteinopathy, including mutations in *TARDBP* itself and also in other genes like *C9ORF72*, progranulin (*GRN*) and others [29, 30]. Interestingly, TDP-43 pathology is mainly absent in familial ALS due to mutations in superoxide dismutase 1 (*SOD1*) [31] and fused in sarcoma (*FUS*) [32].

The term “secondary TDP-43 proteinopathy” refers to the detection of pathological species of TDP-43 in other neurodegenerative diseases, in which TDP-43 is expected to play a part, either pathogenic or reactive, in response to the corresponding primary proteinopathy. This phenomenon is not exclusive to TDP-43, with example of amyloid beta and alpha-synuclein co-pathology in dementia with Lewy bodies (DLB) [33]. Secondary TDP-43 pathology occurs in AD [34], chronic traumatic encephalopathy [35], DLB [36] and Huntington’s disease (HD) [37], etc.

Additionally, TDP-43 proteinopathy has been found in up to 24% of cognitively normal aging brains [38], especially in the oldest population (over 90 years), and located in the amygdala [39]. The amygdala is a key structure for behavior, expression and interpretation of emotions, and eating habits [40], all of which are potentially impaired in FTD and FTD-ALS [41], and it is not surprising that the amygdala and other limbic regions (such as the insular cortex and the hippocampus) are common sites of deposit of TDP-43 proteinopathy [42].

The classification of neurodegenerative disorders based on proteinopathies faces the great challenge of clinical and neuropathological overlap. For example, considering FTD, there are some predictable relationships between the clinical phenotype and the pathological substrate throughout phenotypic development, such as parkinsonism in tau-FTD or semantic disorder in FTD-TDP [43]. However, FTD-TDP and FTD-tau may be clinically indistinguishable, with overlapping behavioral and language impairment [44]. On the other hand, TDP-43 proteinopathies can express a variety of motor, cognitive, and behavioral clinical features, known as the “FTD-ALS spectrum” [45].

In summary, we believe that the great heterogeneity of TDP-43 proteinopathies, together with the lack of a specific signature of their disease pathophysiology, strongly calls for the development of new, accurate, in vivo protein-based biomarkers, with the aim of settling an optimal clinical classification, an earlier diagnosis for patients and, eventually, the start of new research on disease-modifying therapies for TDP-43 proteinopathies.

## Current strategies for in vivo detection of TDP-43 proteinopathy

Currently, TDP-43 proteinopathy remains a post-mortem diagnosis. The detection of TDP-43 depends on the assay used for the different protein species (Fig. 1b). Consequently, N-terminal antibodies might detect aggregates of full-length TDP-43, while C-terminal antibodies are able to recognize cleaved cytotoxic fragments as well [46] (Fig. 1b). Recognition of pTDP-43 is also available via antibodies against phosphorylated serines 409/410 [47].

**Fig. 2:**
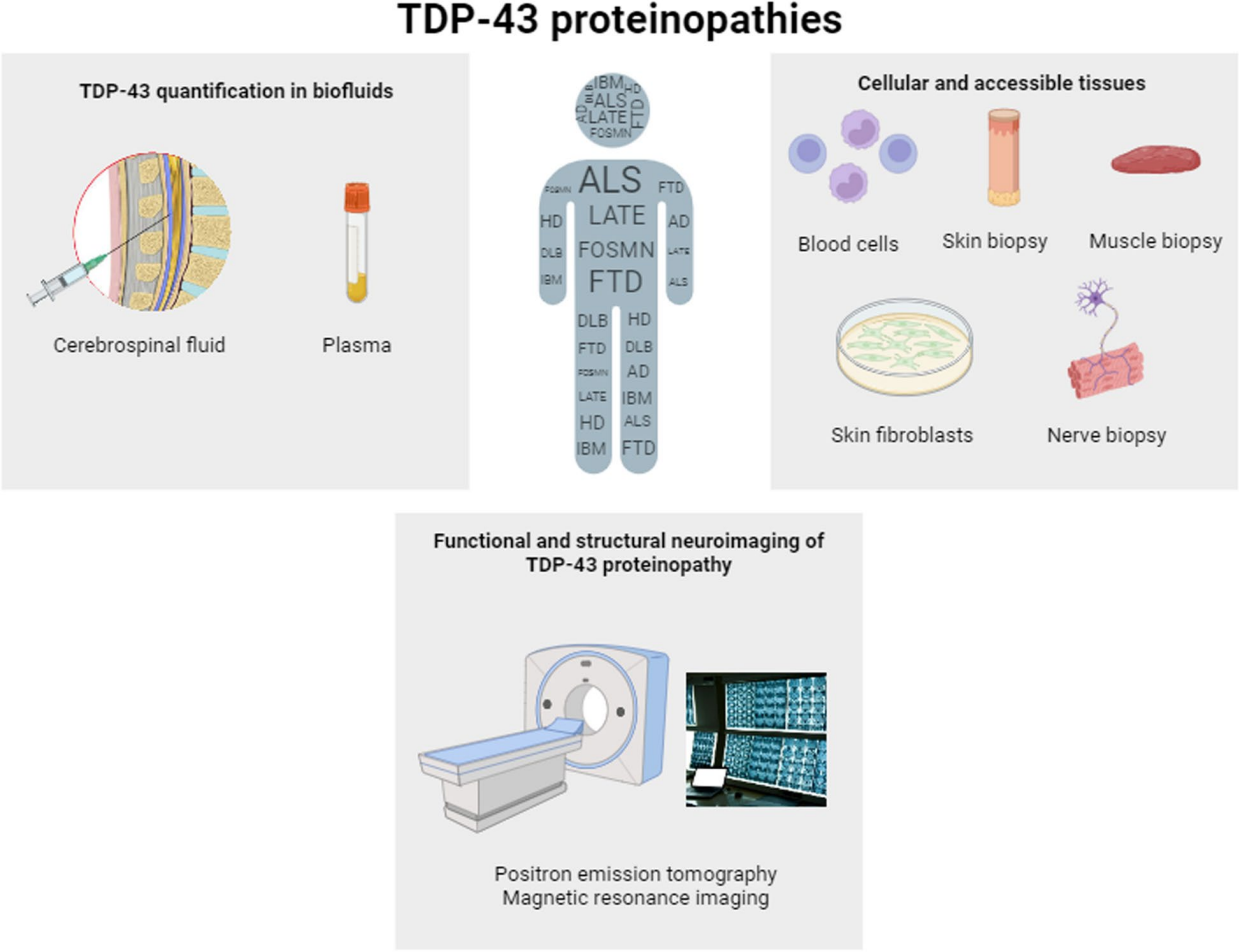
An overview of the different TDP-43 detection approaches considered for diagnostic use, including quantification of free TDP-43 in biofluids (i.e., plasma/serum and CSF) and extracellular vesicles, structural and functional neuroimaging with magnetic resonance imaging and positron emission tomography, and detection of TDP-43 pathology in cells and tissues outside the CNS (blood cells, skin fibroblasts), and other tissues, such as muscle and nerve

Given the unavailability of direct access to neuronal tissue for biopsies and the need for early detection markers, researchers are exploring various avenues to identify TDP-43 proteinopathy in vivo from early disease stages, including brain imaging, CSF analysis, blood samples, and examination of extraneuronal cells and tissues [48] (Fig. 2).

### TDP-43 quantification in biofluids

Classically, two main approaches to detecting pathological species of TDP-43 in biofluids have been considered: antibody-based assays and proteomics. Although pathologic deposits of TDP-43 are observable in human post-mortem brains, detection in patient biofluids such as CSF and serum using antibody-based assays has proven challenging. One the one hand, difficulties primarily arise due to the tendency of most antibodies to bind both the pathologic and the physiological forms of TDP-43 [48]. On the other hand, soluble TDP-43 might tend to aggregate and that might alter the amount detected in the soluble fractions that are classically analyzed. Indeed, some groups have found that it is in the insoluble protein fraction and not in the soluble fraction of the samples where TDP-43 levels are higher in ALS patients [49]. Another potential cause of the variation in the measurements of the TDP-43 levels could be that, unlike other neuronal-specific proteins used as biomarkers in biofluids, TDP-43 is a ubiquitously expressed protein. That means that many other cells in the body, even the blood cells themselves, might contribute to the amount of free TDP-43 found in the blood, in response to different types of damage. This could also mean that the levels of TDP-43 might not represent directly the alteration of the CNS alone, but it could also represent systemic or other organ damage. That is why further studies are needed to understand the sources of TDP-43 in biofluids, which will come also from studying the role of TDP-43 outside the nervous systems, which is not that extensively studied. Careful considerations of the role of TDP-43 systematically and in response to body alterations are needed during the analysis of TDP-43 and to help in the interpretation of the results of TDP-43 levels in biofluids.

Those measurements of physiological and pathologic species of TDP-43 have been normally done in the CSF for being considered a direct exudate from the CNS, in the serum and/or plasma, and in the extracellular vesicles (EVs) extracted from serum or plasma. A summary of all the reviewed studies on the detection in biofluids is presented in Table 1.

**Table 1:** Reported studies on TDP-43 pathology immunodetection in biofluids

	**Disease (*n*)**	**Fluids**	**Primary anti TDP-43 or kit**	**Technique**	**Results**
Foulds et al. (2008) [50]	FTD (*n* = 35) AD (*n* = 102) HC (*n* = 85)	Plasma	Abnova® H00023435-M01 (monoclonal 2E2-D3) -BC001487, ProteinTech Group	ELISA	Elevated levels of TDP-43 protein in 46% patients with FTLD and 22% patients with AD, compared to 8% of control
Kuiperij et al. (2010) [51]	sIBM (*n* = 31), PM (*n* = 48), DM (*n* = 24) HC (*n* = 33)	Plasma	Nonspecified, after Foulds et al. (2008)	ELISA	TDP-43 levels are significantly increased in sIBM, PM and DM plasma as compared to controls
Verstraete et al. (2012) [50]	ALS (*n* = 219) HC (*n* = 100)	Plasma	Abnova® H00023435-M01 (monoclonal 2E2-D3) -BC001487, ProteinTech Group	ELISA	TDP-43 is significantly increased in patients with ALS and positively correlates with age in patients and controls
Ichikawa et al., (2019) [52]	Depression, elderly (*n* = 74) HC (*n* = 58)	Serum	Proteintech® 10782–2-AP Proteintech® 60019–2-Ig	ELISA	TDP-43 is significantly higher in late-life depression patients. This suggests that some depression patients may be in a prodromal stage of FTD or in a very early stage of FTD comorbid with depression
Modgil et al. (2020) [53]	ALS (*n* = 89) HC (*n* = 98)	Plasma	ELISA kit for TDP-43 (Qayee Bio-Technology Co.)	ELISA	ALS patients have significantly lower levels of TDP-43
Ren et al. (2021) [54]	ALS (*n* = 69) HC (*n* = 59)	Plasma	Proteintech® Human TDP-43, KE00005 pTDP-43 ELISA Kit, E9442h EIAab	ELISA	TDP-43 and pTDP-43 levels are significantly higher in ALS. The pTDP-43/TDP-43 ratio is significantly higher in HCs
Bourbouli et al. (2021) [55]	FTD (*n* = 56) ALS (*n* = 58) FTD-ALS (*n* = 16)	Plasma	Human TAR DNA-binding protein 43 ELISA kit; Cusabio Biotech	ELISA	TDP-43 does not differ between FTD and ALS or ALS-FTD
Katisko et al. (2022) [56]	FTD (*n* = 254) HC (*n* = 105)	Plasma	Simoa™ TDP-43 Kii	ELISA	Total levels of TDP-43 in the serum are decreased especially in FTD patients with the *C9orf72* repeat expansion or FTD-MND phenotype
Sampedro et al. (2022) [57]	HD (*n* = 36)	Plasma	Simoa™ TDP-43 Advantage kit	ELISA	TDP-43 levels also reflect cortical thinning and microstructural degeneration, especially in frontal and anterior-temporal regions, which can be correlated with the severity of cognitive, motor and behavioral symptoms
Jamerlan et al. (2023) [58]	SD (*n* = 16), MCI (*n* = 18), EOAD (*n* = 32), LOAD (*n* = 16), and PD (*n* = 12) patients	Plasma	Proteintech® Human TDP-43 #10782–2-AP (NTD) #12892–1-AP (CTD)	ELISA; multimer detection system	A significant increase in pTDP-43 concentrations in patients with SD compared to other neurodegenerative disorders and normal controls
Kojima et al. (2021) [59]	ALS (*n* = 75)	Plasma CSF	Simoa™ NF-light assay, TDP-43 assay, and Human Total Tau assay kits	ELISA	Negative relationship between CSF NfL and TDP-43. Negative correlation between plasma TDP-43 and split hand index
Kasai et al. (2009) [60]	ALS (*n* = 30) HC (*n* = 13) Controls with various neurological disorders (disease controls, *n* = 16)	CSF	Anti-TDP-43 monoclonal antibody (H00023435-M01, clone 2E2-D3, Abnova Corporation, Walnut, USA) Anti-TDP-43 rabbit polyclonal antibody (10782-2-AP, ProteinTech Group, Chicago, USA)	ELISA	The levels of TDP-43 in the CSF are significantly higher in ALS patients than in the age-matched controls, especially in the first 10 months of onset.,
Noto et al. (2011) [61]	ALS (*n* = 27) Controls with various neurological disorders (disease controls, *n* = 50)	CSF	Anti-TDP-43 monoclonal antibody (H00023435- M01, clone 2E2-D3, Abnova Corporation, Walnut, USA Anti-TDP-43 rabbit polyclonal antibody (10782-2-AP, ProteinTech Group, Chicago, USA)	ELISA	CSF TDP-43 levels are increased only in ALS patients. Lower CSF TDP-43 levels may be associated with shorter survival time
Hosokawa et al. (2014) [62]	ALS (*n* = 13) GBS (*n* = 7)	CSF	Anti-TDP-43-N-ter monoclonal antibody, clone 2E2-D3 (Abnova Corp., Taipei), for capture Anti-TDP-43 N-ter rabbit polyclonal antibody (catalog code 10782-2-AP, ProteinTech Group Inc., Chicago, IL) for detection	ELISA	TDP-43 concentrations in the CSF are significantly higher in ALS than in GBS
Hu et al. (2013) [63]	First validation cohort (*n* = 30): 10 subjects (FTLD-TDP *n* = 3) with normal p/t-Tau, 20 subjects (FTLD-TDP *n* = 16) with decreased p/t-Tau Second validation cohort (*n* = 100): 61 subjects (FTLD-TDP *n* = 6) with normal p/t-Tau and 39 subjects (FTLD-TDP *n* = 27) with decreased p/t-Tau	CSF	Aβ42, t-Tau, and p-Tau181: commercially available kits (AlzBio3; Innogenetics, Ghent, Belgium) Levels of other candidate CSF FTLD-TDP biomarkers were measured by modifying commercially available immunoassays: Multiplex assay: Agouti-related peptides and adrenocorticotropic hormones (Millipore, Billerica, MA) Singleplex assays: Eotaxin-3 (Millipore), Fas (Affymetrix/Procarta, Santa Clara, CA), and IL-23 (R&D Systems, Minneapolis, MN). IL-17 measurements were tried in 5 commercially available kits (Millipore; Life Technologies, Grand Island, NY; Affymetrix/Procarta; R&D Systems; and Affymetrix/eBioscience, San Diego, CA) with no reliably detectable levels. IL-17 in the original biomarker panel was thus replaced by IL-23	Commercial immunoassays	First validation cohort: FTLD-TDP cases have decreased levels of p-tau181 and IL-23, and increased Fas Second validation cohort: CSF p-tau181/total tau ratio < 0.37 distinguishes FTLD-TDP from FTLD-tau, AD, and healthy seniors with 82% sensitivity and 82% specificity Reduced CSF p-tau181/total tau ratio represents a reproducible, validated biomarker for FTLD-TDP with performance approaching well-established CSF AD biomarkers
Borroni et al. (2015) [64]	FTLD (*n* = 79)	CSF	ELISA test (Innotest hTau Antigen kit and Innotest Phospho-Tau (181P); Innogenetics, Ghent, Belgium)	ELISA	Significantly reduced CSF p/t-Tau ratio in FTLD-TDP relative to FTLD-Tau
Junttila et al. (2016) [65]	FTLD (*n* = 69) ALS (*n* = 21) There were 30 patients with the *C9ORF72* expansion and 60 patients without the expansion	CSF	TDP-43 levels: commercial ELISA (Cusabio, China) Aβ1-42, t-tau, and phospho-tau levels: commercial ELISA (Innogenetics, Ghent, Belgium)	ELISA	CSF TDP-43 levels show no variance between *C9ORF72* expansion carriers and noncarriers. Levels are higher in ALS than in FTLD patients, regardless of the *C9ORF72* status Additionally, males have notably higher TDP-43 levels than females
Kuiperij et al. (2017) [66]	Ventricular CSF: FTD (*n* = 29), AD (*n* = 20), non-demented controls (*n* = 19) Lumbar CSF of FTD-TDP (*n* = 36) and FTD-tau (*n* = 7)	Lumbar and ventricular CSF	Total TDP-43: - Goat polyclonal antibody directed against TDP-43 (Abcam, Cambridge, UK) - Rabbit polyclonal antibody directed against TDP-43 (Proteintech Europe, Manchester, UK) - INNOTEST -Amyloid(1–42) ELISA kit; Fujirebio, Ghent, Belgium pTDP-43: - Rat polyclonal antibody directed against TDP-43 phosphorylated at Ser409/Ser410 (Merck Millipore, Billerica, MA, USA) - Biotinylated rabbit polyclonal antibody directed against TDP-43, diluted in reagent diluent (R&D systems Europe, Abingdon, UK) Total tau and p-tau proteins: - Innotest: total tau and p-tau181ELISA kits (Fujirebio)	ELISA	In ventricular CSF, t-TDP-43 and t-tau levels are significantly different between FTLD-tau and FTLD-TDP cases In a pilot study using lumbar CSF, the p-tau/t-tau ratio, but not t-TDP-43 level, is significantly different between FTLD-TDP and FTLD-tau patients CSF tau, rather than TDP-43 proteins, may have diagnostic value in the differentiation of FTLD patients with either tau or TDP-43 pathology
Bourbouli et al. (2017) [67]	ALS (*n* = 32) FTD (*n* = 51) HC (*n* = 17)	CSF	TDP-43: Human TAR DNA-binding protein 43 ELISA kit; Cusabio Biotech Co., Ltd., China. Aβ42, total tau, p-tau181: “β-amyloid 1–42,” “Innotest hTau antigen,” and “p-tau181,” respectively; Fujirebio, Gent, Belgium	ELISA	Both ALS and FTD patients present with higher TDP-43 and total tau levels compared to the control group. The combination of biomarkers TDP-43 × total tau / total p-tau181 achieved the best discrimination between ALS or FTD and controls
Khosla et al. (2020) [68]	SALS (*n* = 54) HC (*n* = 32)	CSF	Commercial ELISA Kit (Qayee Biological Technology, Shanghai, China)	ELISA	No significant differences in TDP-43 CSF levels between ALS and HC
Sproviero et al. (2018) [69]	SALS (*n* = 30) HC (*n* = 36)	Plasma EV	Western Blot kit (BioRad, Italy). Anti-TDP-43 (Proteintech)	Western Blot	EVs from ALS patients are enriched with TDP-43 and pTDP-43
Zhang et al. (2020) [70]	AD (*n* = 24) HC (*n* = 15)	NDEV	ELISA kits for TDP-43 (Signalway Antibody) and CD81 for normalization (Cusabio)	ELISA	Higher normalized plasma NDEV concentrations of TDP-43 in AD compared to HC. No relationship between TDP-43 levels and behavioral or motor symptoms among AD patients

*ALS* Amyotrophic lateral sclerosis, *DM* Dermatomyositis, *EOAD* Early-onset Alzheimer’s disease, *FALS* Familial ALS, *FTD* Frontotemporal dementia, *GBS* Guillain-Barré syndrome, *HC* Healthy controls, *HD* Huntington’s disease, *LOAD* Late-onset Alzheimer’s disease, *NC* Neurological controls (patients with other neurological diseases), *NC* Stable normal control, *NDEV* Neuronal-derived extracellular vesicles, *MCI* Mild cognitive impairment, *PM* Polymyositis, *SALS* Sporadic ALS, *SD* Semantic dementia (semantic variant of primary progressive aphasia), *sIBM* Sporadic inclusion body myositis

#### Plasma and serum

Early attempts to detect abnormal TDP-43 levels in plasma aimed to potentially distinguish between FTD-TDP and FTD-tau, considering AD patients as a neurological control for TDP-43 proteinopathy. Foulds et al. used enzyme-linked immunosorbent assay (ELISA) with monoclonal anti-TDP-43 recognizing the NTD. Their results revealed significantly elevated average TDP-43 levels in both FTD and AD patients compared to healthy controls, regardless of age or disease onset [71]. Further research from the same group found a positive, no significant trend between plasma pTDP-43 levels and FTD-TDP compared to FTD-tau patients with confirmed histopathology [50]. Ichikawa et al. found increased levels of TDP-43 in elderly with late-life depression compared to age-matched controls, suggesting that some patients with depression might be in a prodromal state of FTD [52]. In one study, FTD patients carrying either *C9ORF72* repeat expansions or *GRN* mutations exhibited higher levels of pTDP-43 in plasma compared to other patients diagnosed with FTD and to healthy controls [72]. Conversely, another study measured soluble TDP-43 in serum using a more sensible system, the Simoa® TDP-43 kit, reporting slightly decreased TDP-43 levels in FTD-TDP compared to FTD-tau and healthy controls [56].

For ALS patients, some studies replicated methods by Foulds et al. in plasma compared to healthy controls, finding a significant increase of TDP-43 levels compared to controls, as well as a positive correlation with age, in both patients and controls [54, 73]. The relationship between TDP-43 and age is further discussed in this review (section “Upcoming challenges”). The average TDP-43 levels showed correlation to clinical progression [59], but no changes were found across the FTD-ALS spectrum phenotypes [55]. On the other hand, another study in Indian patients using immunodetection by ELISA found exactly the opposite, that is, lower levels of TDP-43 in ALS patients compared to healthy controls [53]. The authors suggested that the TDP-43 levels can be lower in plasma as the protein is sequestered inside cells.

The same methods were applied to identify other primary or secondary TDP-43 proteinopathy in different neurodegenerative diseases. Jamerlan et al. claimed higher plasma TDP-43 levels in semantic variant of primary progressive aphasia compared to healthy and neurological controls [58]. Plasma TDP-43 levels in both IBM and other inflammatory myopathies (polymyositis and dermatomyositis) were significantly higher compared to healthy controls, though data dispersion was remarkable [51]. Sampedro et al. studied plasma TDP-43 levels in a cohort of 36 patients with Huntington’s disease, in which TDP-43 is considered a secondary proteinopathy. They found that increased TDP-43 levels were related to cortical thinning and microstructural degeneration, especially in frontal and anterior temporal regions, which could correlate to the severity of cognitive, motor and behavioral symptoms [57].

#### CSF

The initial investigations of TDP-43 in the CSF as a biomarker for ALS used the same anti-TDP-43 antibody recognizing the NTD, showing a significant increase of total TDP-43 concentration [60]. Noto et al. used a monoclonal anti-TDP-43 targeting the CTD in the CSF of ALS patients showed increased TDP-43 in ALS compared to controls. Interestingly, this study associated lower levels of TDP-43 with less survival time [61]. A small study in ALS and Guillain-Barré syndrome with anti-NTD and anti-CTD antibodies showed increased TDP-43 levels in the CSF of ALS patients [62], reinforcing the relationship between TDP-43 levels in CSF and TDP-43 proteinopathy.

Junttila et al. showed increased levels of TDP-43 in the CSF of ALS and FTD compared to controls, with notably higher levels in males than in females, and no differences between *C9ORF72* carriers and noncarriers [65]. However, the CSF level of pTDP-43 is increased in FTD patients with *C9ORF72* repeat expansions or *GRN* mutations, compared to other FTD patients and healthy controls [72]. By contrast, another study found no significant differences in TDP-43 levels in the CSF samples from a cohort of 54 sporadic ALS patients and 32 controls from northern India [68].

Several studies approached detection of tau and p-tau181 in the CSF as a biomarker of clinical interest to discriminate FTD-tau and FTD-TDP [63, 64, 66]. Unfortunately, this "tau-negative" diagnostic approach is not applicable as a biomarker for the broader spectrum of TDP-43 proteinopathy diseases, and p-tau181 is not specific for frontotemporal lobar degenerations associated with tau, which are also influenced by amyloid deposition [34]. Bourbouli et al. found increased TDP-43 and tau levels in both ALS and FTD compared to healthy controls, and propose a combined index with TDP-43, tau and p-tau181 to improve data dispersion and increase discrimination between ALS-FTD spectrum and healthy controls [67].

In all of the studies reviewed here, the mean total TDP-43 protein concentration was considered as their primary endpoint. However, as there is often significant dispersion of the data, results require cautious interpretation. The variability of TDP-43 and pTDP-43 levels between patients makes it challenging to establish appropriate cut-off values to achieve optimal diagnostic results.

#### EVs

TDP-43 is transmitted across axon terminals inside EVs [74]. Mutant TDP-43 is transported by EVs in *TARDBP* transgenic animal models, and that was suggested to play a role in the pathogenesis of TDP-43 proteinopathy [75] or be a neuronal strategy for aberrant TDP-43 clearance [76].

Studies on *TARDBP* transgenic murine and canine models showed increased levels of TDP-43 and pTDP-43 in plasma-derived EVs compared to controls [77]. In humans, TDP-43 and pTDP-43 have been detected by Western blot in plasma EVs from ALS patients [69]. However, evidence on altered TDP-43 levels in EVs of patients with neurodegenerative diseases remains scarce. One study in AD patients showed increased levels of TDP-43 in plasma neuronal-derived extracellular vesicles (NDEV) compared to healthy controls. In this study, there was no relationship of TDP-43 levels with behavioral or motor symptoms among AD patients, nor with the apolipoprotein E (*APOE*) ε4 genotype [70]. In contrast, one study in ALS patients and controls applying immuno-electron microscopy suggested that pTDP-43 might not be an intravesicular cargo of plasma-derived EV [78]. Another study in patients with LATE neuropathological changes (LATE-NC) found increased TDP-43 in astrocyte-derived EVs (ADEVs) but neither in NDEVs nor in microglial-derived EVs [79], suggesting a pivotal role of astrocytes in the pathogenesis of TDP-43 proteinopathy. However, no correlation was found between ADEV TDP-43 levels and clinical variables such as sex, cognitive status or *APOE* ε4 genotype. More extensive research is needed to clarify the role of human EVs in TDP-43 proteinopathies as a clinical biomarker.

#### Detection of cryptic exon neoepitopes as a read-out of TDP-43 splicing loss of function

An emerging approach to detecting TDP-43 proteinopathy focuses on the characterization of neoepitopes from cryptic exons which are exposed due to the impairment of TDP-43 splicing function caused by TDP-43 nuclear loss of function. A TDP-43-dependent cryptic epitopes, hepatoma-derived growth factor-like protein 2 (HDGFL2), has been recently detected to be increased via sandwich ELISA in CSF samples of different cohorts of *C9ORF72* ALS patients, *C9ORF72* presymptomatic carriers and sporadic ALS patients, compared to healthy individuals and neurologic controls with diagnoses of migraine and normal pressure hydrocephalus (NPH) [80], proposing a novel, specific CSF biomarker for ALS far earlier than neurofilament chains. However, the data showed dispersion within groups, even among controls. In fact, while the mean values of HDGFL2 ELISA signal were significantly higher, many patients and carriers showed normal values, and a few controls, especially from the older (NPH) group, also had higher levels. Further research is paramount in this interesting field, and we support identification of confounders (age, sex) as an essential preliminary step to better understand the results. In this same work, replication of these results in blood samples from C*9ORF72* ALS patients and carriers did not lead to statistically significant results. Similarly, a recent study identified de novo proteins in the CSF of ALS/FTD patients as a result of the translation of several mRNA transcripts harboring cryptic exons, which resulted from the functional loss of TDP-43 in these disorders [81]. These studies open the door to potential new strategies to indirectly measure the function of TDP-43 in the CSF of ALS/FTD patients. Further research is needed to determine the validity of these potential peptides as biomarkers of disease, or the potential application of these strategies in other biofluids or accessible tissues.

### Functional and structural neuroimaging of TDP-43 proteinopathy

#### PET

PET is a useful neuroimaging technique for the diagnosis of many neurodegenerative diseases. A TDP-43 PET radiotracer would be of utmost interest in the clinic for the differential diagnosis of TDP-43 proteinopathies, as the currently used amyloid and tau tracers in AD. Unfortunately, such radiotracers are still unavailable [82].

The most common PET radiotracer is [^18^F]-fluorodeoxyglucose (FDG), which is informative of synaptic function and regional areas of neurodegeneration. However, FDG is not specific, and is unable to distinguish between the different forms of FTD. Some studies in ALS have reported hypometabolism in frontal regions, as theoretically expected, as well as hypermetabolism in posterior regions, compared to healthy controls [83, 84].

FDG-PET of AD patients with and without associated TDP-43 proteinopathy revealed greater hypometabolism in medial temporal, frontal superior medial, and frontal supraorbital regions in TDP-positive cases compared to TDP-negative cases [85]. Grothe et al. recently found distinct temporo-limbic and temporo-parietal FDG-PET signatures in a small cohort of LATE-NC and AD patients, respectively. In a larger cohort of patients with a clinical diagnosis of AD, the patients showing a LATE-NC–like FDG-PET pattern were significantly older, exhibited a predominant amnestic phenotype, had a slower disease course, and showed less abnormal amyloid and tau CSF biomarkers as well as lower prevalence of *APOE* ε4 allele [86]. Therefore, FDG-PET may be a promising predictor of LATE-NC, although further research is needed.

Studies on other PET radiotracers in TDP-43 proteinopathies are scarce. A study of amyloid PET with radiotracer [^18^F]-florbetapir in 30 AD cases with pathological confirmation showed a selective effect of TDP-43 on hippocampal PET signal that appears to be partially dependent on TDP-43 mediated atrophy [87]. Two studies with synaptic vesicle 2A tracers have shown synaptic loss in behavioral variant FTD patients compared to controls, which correlates with behavioral impairment [88, 89].

Tau PET radioligands such as [^18^F]-flortaucipir have proven to detect beta-sheet structure in TDP-43, and one in vivo study showed that this radiotracer mirrored the expected distribution of TDP-43 pathology in patients with semantic variant of primary progressive aphasia [90]. Based on these principles, a number of tau-PET radioligands were assayed in ALS post-mortem tissues, though no colocalization to pTDP-43 immunohistochemistry was found [91]. In this line, a study with older individuals showed that TDP-43 pathology does not affect [^18^F]-flortaucipir uptake [92].

The development of a specific radiotracer for TDP-43 would enable in vivo investigation in physiological aging and disease. Various groups are currently working in the development of TDP-43 radiotracers, so advancement in the in vivo imaging of TDP-43 proteinopathies might be just around the corner.

#### Structural neuroimaging related to TDP-43 proteinopathy patterns of degeneration

Similar to FDG-PET, structural CNS neuroimaging in neurodegenerative disorders is a valuable and accessible tool to detect areas of cortical structural pathology. Brain magnetic resonance imaging (MRI) may show white matter T2 hyperintensity in corticospinal tracts but with low diagnostic accuracy [93]. Another group explored diffusion-tensor imaging (DTI) in ALS as a proxy to evaluate the integrity of white matter fibers, finding fractional anisotropy at the brainstem as a differential parameter in ALS compared to healthy controls, proposing DTI MRI as a clinical biomarker for ALS [94].

Cortical atrophy in FTD mainly involves anterior cingulate and frontoinsular cortex, striatum and amygdala, sparing posterior cortex and especially precuneus [95]. Patients carrying pathologic variants of *TARDBP* gene (for TDP-43) are associated with increased rates of atrophy in the hippocampus, temporal pole and middle frontal gyrus, while FTD-TDP and AD-related TDP-43 proteinopathy are also associated with increased rates of atrophy in the inferior temporal lobe and amygdala [96]. Deep analysis of MRI data, such as grey matter maps, found some distinctive atrophy patterns between Pick’s disease (FTD-tau), FTD-FUS and FTD-TDP [97], proposing a MRI-based predictive model.

Hippocampal atrophy in CA1 and subiculum areas has also been associated with hippocampal sclerosis of aging [98], often related to LATE-NC [34]. The TDP-43 burden is uniquely associated with inward deformation in bilateral CA1 and subiculum, controlled for the effects of beta-amyloid and tau pathology [99]. On the contrary, in non-amnestic variants of AD the presence of TDP-43 does not correlate with memory loss or hippocampal atrophy, enhancing the influence of TDP-43 proteinopathy on memory impairment in AD and LATE-NC [100].

### Detection of TDP-43 in accessible cells and tissues

TDP-43 is ubiquitously expressed and distributed across multiple tissues and cell types beyond the CNS [13]. Pathological species of TDP-43 might be found in different cell types and tissues, reflecting early disease stages in the CNS. There is increasing evidence supporting that the TDP-43 pathology causes not only neurodegeneration, but also alterations in bioenergetic metabolism outside the CNS, suggesting a multisystem disorder [101] which remains poorly understood. This widespread distribution enables the detection of TDP-43 pathology in cell types beyond the CNS (Fig. 2) and can be used for early diagnosis and/or as a biomarker of progression of disease. Furthermore, cell analysis allows exploration of other pathological features, especially in relation to the subcellular location, such as cytoplasmic/nuclear location ratio, that could contribute to classification of patients [17]. Reviewed studies are summarized in Table 2.

**Table 2:** Reported studies on extra-neural TDP-43 pathology immunodetection

	**Disease (*n*)**	**Cell type**	**Primary anti-TDP-43**	**Target/Epitope**	**Results**
Suzuki et al*.* (2010) [102]	SALS (*n* = 15) HC (*n* = 15)	Skin biopsy	Sigma®, “polyclonal"	(Not reported)	Increased % TDP-43^+^ cells in ALS vs HC Increased % TDP-43^+^ cells correlates with ALS progression
Sorarù et al. (2010) [103]	ALS (*n* = 30) HC (*n* = 30)	Muscle biopsy	Not reported	(Not reported)	Nuclear deposits of TDP-43 in patients and controls. No evidence of TDP-43 cytoplasmic translocation nor presence of truncated C-terminal fragments
De Marco et al*.* (2011) [104]	*TARDBP*-ALS (*n* = 4) *SOD1*-ALS (*n* = 1) ALS, other (*n* = 12) HC (*n* = 13)	Monocytes (PBMC)	Abnova® H00023435-M01 (monoclonal 2E2-D3)	CTD	Total TDP-43 unchanged. TDP-43 translocation in SALS and TARDBP-ALS. TDP-43 translocation absent in SOD1
Nardo et al*.* (2011) [105]	ALS (*n* = 94) NC (*n* = 23) HC (*n* = 41)	PBMC	Not reported	(Not reported)	TDP-43 associated with disease progression in a longitudinal study
Pinkus et al*.* (2014) [106]	sIBM (*n* = 13) DM (*n* = 3) PM (*n* = 3) Muscular dystrophy (*n* = 3) ALS (*n* = 2) Non-neuromuscular disease controls (*n* = 2)	Muscle biopsy	Proteintech® 10782–2-AP	NTD	Myonuclear depletion of TDP-43 in sIBM muscle. TDP-43 sarcoplasmic aggregates. No data of TDP-43 aggregation for included ALS samples
Kierdaszuk et al*.* (2015) [107]	sIBM (*n* = 8) LGMD (*n* = 3) DM2 (*n* = 1)	Muscle biopsy	Abnova® (unspecified)	(Not reported)	Pathological deposits of TDP-43 in all sIBM as well as in control cases
Wang et al*.* (2015) [108]	TARDBP-ALS (not reported) HC (not reported)	Skin biopsy	Not reported	(Not reported)	Increased TDP-43^+^ skin cells in TARDBP-ALS vs HC
Paré et al*.* (2015) [109]	SALS (*n* = 6) *C9orf72*-ALS (*n* = 1) *C9orf72*-carriers (*n* = 5) HC (*n* = 5)	Skin fibroblasts Engineered skin tissues	Proteintech® 12892–1-AP	CTD	Increased TDP-43 aggregates in ALS vs HC tissues. TDP-43 not increased in *C9orf72*-ALS fibroblasts
Yang et al*. (*2015) [110]	*TARDBP*-ALS (*n* = 2) *UBQLN2*-ALS (*n* = 1) HC (*n* = 4)	Skin fibroblasts	Proteintech®, “polyclonal"	(Not reported)	Cytoplasmic TDP-43 aggregation in cells from three SALS, two TDP-43 M337V and one iUBQLN2-T487I patient
Alquézar et al*.* (2016) [111]	(Cell line)	*GRN* ^-/-^ lymphocytes	Proteintech® 10782–2-AP Proteintech® 22309–1-AP	NTD pTDP-43 (S409/410)	Increased pTDP-43^+^ cells in *GRN* ^−/−^ lymphocytes cytoplasmic TDP-43 not increased
Orrù et al*.* (2016) [112]	ALS (not reported) HC (not reported)	Skin fibroblasts	Proteintech® 10782–2-AP Proteintech® 12892–1-AP Proteintech® 22309–1-AP	NTD CTD pTDP-43 (S409/410)	Loss of nuclear TDP-43
Abe et al*.* (2017) [113]	SALS (*n* = 22) NC (*n* = 26) ALS autopsies (*n* = 3)	Skin biopsy	Proteintech® 12892–1-AP	CTD	Increased TDP-43^+^ cells in ALS vs HC
De Marco et al*.* (2017) [114]	*TARDBP*-ALS (*n* = 4) *C9ORF72*-ALS (*n* = 4) *SOD1*-ALS (*n* = 6) *FUS*-ALS (*n* = 2) *VCP*-ALS (*n* = 2) FALS, unknown (*n* = 1) SALS (*n* = 5) HC (*n* = 13)	Monocytes (PBMC)	Abnova® H00023435-M01 (monoclonal 2E2-D3)	CTD	Cytoplasmic TDP-43 translocation in SALS and *TARDBP-*, *FUS-* and *VCP-*FALS, but absent in *SOD1-* and *C9ORF72-*FALS
Ren et al*.* (2018) [54]	SALS (*n* = 18) HC (*n* = 18)	Skin biopsy: intraepidermal fibers	Not reported	(Not reported)	pTDP-43^+^ in 33% ALS and 5.56% controls
Posa et al*.* (2018) [115]	SALS (*n* = 7) *C9ORF72*-ALS (*n* = 1) HC (*n* = 6)	Monoclonal immortalized lymphocytes	Proteintech® 10782–2-AP Proteintech® 22309–1-AP	NTD pTDP-43 (S409/410)	Total TDP-43 unchanged in ALS vs HC; Cytoplasmic TDP-43 translocation in ALS vs HC; Increased pTDP-43^+^ in ALS vs HC
Codron et al*.* (2018) [116]	SALS (*n* = 6) HC (*n* = 4)	Skin fibroblasts	Proteintech® 10782–2-AP	NTD	No differences between ALS and HC. Results discourage the use of TDP-43 as a biomarker for ALS
Fourier et al. (2019) [117]	FTD (*n* = 9)	Platelets	Proteintech® 10782–2-AP Proteintech® 12892–1-AP	NTD CTD	Pilot study: suitability for detection of TDP-43 in platelets from blood samples of FTD patients by means of Simple Western® approach
Luotti et al. (2020) [49]	ALS (*n* = 93) NC (*n* = 111) HC (*n* = 104)	PBMC	Proteintech® 12892–1-AP	CTD	Increased insoluble fraction of TDP-43 in ALS compared to HC and NC. Positive linear correlation with duration of symptoms. No association with survival PBMC TDP-43 cannot be used as a single parameter to accurately distinguish ALS patients from controls
Riancho et al*.* (2020) [118]	SALS (*n* = 8) HC (*n* = 4)	Skin fibroblasts	Proteintech®	(Not reported)	Cytoplasmic aggregates of TDP-43 in ALS. Higher susceptibility to DNA damage in ALS vs HC
Romano et al*.* (2020) [119]	SALS (*n* = 2) *TARDBP*-ALS (*n* = 4) HC (*n* = 2)	Skin fibroblasts	Proteintech®, “monoclonal”	(Not reported)	TDP-43 cytoplasmic translocation under oxidative stress conditions
Riva et al. (2022) [120]	ALS (*n* = 71) Non-ALS mimics (*n* = 31)	Motor nerve biopsy	Proteintech® 10782–2-AP Proteintech® 22309–1-AP	NTD pTDP-43 (S409/410)	Significantly increased TDP-43 and pTDP-43 in both axons and Schwann’s cells, even prior to axonal degeneration
Kurashige et al. (2022) [121]	First step: - SALS (*n* = 10) - NC (*n* = 12) Second step: cohort of 450 patients undergoing muscle biopsy for diagnostic purpose	Muscle biopsy (intramuscular nerve bundles)	CosmoBio® TIP-PTD-M01 Proteintech® 22309–1-AP	pTDP-43 (S409/410)	Accumulation of pTDP-43 in intramuscular nerve bundles of ALS patients, which was absent in controls. Patients from the cohort exhibiting pTDP-43 aggregation (*n* = 33) were confirmedly diagnosed with ALS
Rubio et al*.* (2022) [122]	SALS (*n* = 44) NC (*n* = 10) HC (*n* = 10)	Skin biopsy	Proteintech® 12892–1-AP	CTD	Increased %TDP-43^+^ in ALS vs HC and NC
Liu et al*.* (2022) [123]	FOSMN (*n* = 6)	Skin fibroblasts Muscle biopsy	Proteintech®, “polyclonal"	(Not reported)	Cytoplasmic aggregates of TDP-43 in both fibroblasts and muscle cells in FOSMN
Quek et al*.* (2022) [124]	SALS (*n* = 30) HC (*n* = 20)	Monocyte-derived iPSC-derived microglia	CosmoBio® #TIP-TD-P09	CTD	Cytoplasmic aggregates of TDP-43 and pTDP-43

*ALS* Amyotrophic lateral sclerosis, *CTD* C-terminal domain, *DM2* Myotonic dystrophy type 2, *FALS* Familial ALS, *FOSMN* Facial onset sensory and motor neuropathy, *HC* Healthy controls, *LGMD* Limb-girdle muscular dystrophy, *NC* Neurological controls (patients with other neurological diseases), *NTD* N-terminal domain, *PBMC* Peripheral blood mononuclear cells, *SALS* Sporadic ALS, *sIBM* Sporadic inclusion body myositis

#### Blood cells

A few successful attempts to differentiate nucleus *versus* cytoplasmic levels have been conducted using cells from the blood of patients and healthy controls. An increase of cytoplasmic accumulation of TDP-43 in isolated peripheral blood mononuclear cells (PBMCs) has been found in *TARDBP*-ALS patients (A382T, G368S) and in about 50% of cases of sporadic ALS compared to controls. As expected, no significant differences were found between *SOD1*-ALS patients and controls. Interestingly, the total quantification of TDP-43 from cell lysates showed no differences between ALS patients and controls [104]. The same group reported PBMCs from patients with sporadic ALS and familial ALS with mutations in *TARDBP*, *FUS* and valosin-containing protein (*VCP*) showed cytoplasmic TDP-43 translocation, whereas this translocation was absent in familial ALS carrying mutations in *SOD1* and *C9ORF72* repeat expansions [114]. In a longitudinal study with ALS patients compared to healthy and neurological controls, the quantity of TDP-43 in PBMCs was associated with disease progression [105]. In another study with a large cohort of 93 patients with ALS, analysis of soluble and insoluble fractions of TDP-43 in PBMCs showed an increased insoluble fraction of TDP-43 compared to healthy and neurologic controls, with a positive linear correlation with duration of symptoms, but no association with survival. Authors recognized that TDP-43 as a single biochemical parameter was not able to accurately distinguish ALS patients from controls, and several combinations of PBMC levels of insoluble TDP-43 along with other proteins were proposed, such as soluble peptidyl-prolyl cis–trans isomerase A (PPIA) and hnRNPA2B1 [49].

Two studies measured TDP-43 in lymphoblastoid cell lines immortalized from patients with ALS and FTD due to *GRN* mutations, and observed increased cytoplasmic TDP-43 levels compared to healthy controls, with no differences in total TDP-43 quantity [111, 115]. In these studies, the cell pattern depicts the typical translocation of TDP-43 commonly found in FTD-TDP and ALS.

A more sophisticated approach for TDP-43 quantification in blood cells is the use of PBMC-derived induced pluripotent stem cells (iPSCs). Using this model, Quek et al. found abnormal cytoplasmic inclusions positive for TDP-43 and/or pTDP-43 in iPSC-derived microglia obtained from blood monocytes of patients with ALS, while the cytoplasmic inclusions were absent in cells from healthy controls [124].

One study involving platelets found a significantly higher TDP-43 concentration in ALS patients compared to healthy controls, as quantified by ELISA. Nevertheless, the utilization of these concentrations as a definitive biomarker for ALS is limited due to the presence of overlapping values between a subset of ALS patients and control individuals [125]. Another study developed an automated capillary nano-immunoassay (Simple Western®) to quantify total TDP-43 in platelets from 9 individuals with *C9ORF72* + behavioral variant of FTD. By using an anti-NTD antibody and confirming with anti-CTD, results showed a good quantitative performance but also large inter-individual variations beyond those attributable to the technique, calling for further confirmation in larger cohorts of patients [117].

#### Skin and fibroblasts

The skin has been a strong research topic in ALS for more than a century, dating back to Jean-Martin Charcot's observations that ALS patients experienced fewer bedsores than other bedridden patients, although this fact was ultimately refuted by further evidence [126]. Multiple research endeavors have focused on skin biopsies from patients with ALS compared to healthy controls, with a trending increased proportion of cells exhibiting TDP-43 inclusions (“TDP-43-positive” cells) in ALS patients compared to controls [102, 113, 122]. Additionally, a consistent finding across all studies is the correlation between the proportion of “TDP-43-positive” cells and the disease duration in individuals afflicted with ALS.

Loss of nuclear TDP-43 and increased cytoplasmic TDP-43 have been found in fibroblasts isolated in skin biopsies of ALS patients [108, 110, 112, 118, 119] and by means of more complex approaches such as developed tissue-engineered skins [109]. Ren et al. showed increased pTDP-43 inclusions in intraepidermal fibers from skin biopsies from patients with ALS compared to controls. Furthermore, cytoplasmic aggregates of TDP-43 were also found in skin fibroblasts of a small cohort of patients with FOSMN, strengthening the link of FOSMN to ALS [123]. Controversially, only one single study did not find significant differences in TDP-43 aggregates between ALS and healthy controls in skin fibroblasts, discouraging the use of skin TDP-43 as a biomarker for ALS [116].

Research evidence on TDP-43 detection in the skin of FTD patients is sparse. Leskelä et al. did not find differences in cytoplasmic TDP-43 between FTD and controls [127]. This is not surprising, as about 40%–50% of FTD patients are expected to be negative for TDP-43 pathology (accounting for tau and FUS pathology). Other studies found specific proteins related to specific familial FTD-ALS variants in skin cells, such as VCP [128] and FUS [129].

#### Muscle

The detection of TDP-43 alterations in muscle tissue has been a subject of interest in those TDP-43 proteinopathies directly related to neuromuscular diseases, especially ALS and IBM, highlighting a potential pathological role of TDP-43 in skeletal muscle [103].

Sorarù et al. carried out Western blot and immunohistochemical procedures on muscle biopsies from 30 ALS patients and 30 healthy controls. They found only nuclear TDP-43 in both groups, with no Western blot ~ 25 kDa bands attributable to C-terminal fragments. Thus, the authors discouraged the use of TDP-43 as a biomarker outside the CNS [106]. However, there is no further information on the anti-TDP-43 antibody used in these assays. It is not expected that anti-NTD antibodies would recognize cytoplasmic C-terminal fragments. We consider that this approach should be replicated with more extensive methods and other primary antibodies (anti-CTD, anti-pTDP-43) before rejecting muscle biopsy as a TDP-43-based in vivo biomarker for ALS.

TDP-43 cytoplasmic deposits are found in muscle samples of the aforementioned cohort of patients with FOSMN, in addition to the detection of the deposits in fibroblasts as reviewed above [123]. There are two other studies assessing TDP-43 in vivo, in muscle biopsies of IBM patients, revealing nuclear depletion of TDP-43 and other hnRNPs [107], and TDP-43 cytoplasmic deposits [130]. Although these studies show promising results for the potential use of TDP-43 alterations in muscle biopsy as a biomarker in vivo, they need to be further replicated, with larger number of patients and appropriate controls. Thus, the detection of pathological alterations of TDP-43 in muscles in vivo remains to be elucidated, especially in TDP-43 proteinopathies associated with muscle dysfunction.

Regarding the description of TDP-43 pathology in muscles in post-mortem examinations, a few studies focused on pTDP-43 aggregation in skeletal and cardiac muscles by comparing post-mortem muscle tissues of ALS patients to muscle biopsies of non-ALS patients with neurogenic atrophy as controls. One study found sarcoplasmic deposits of pTDP-43 in a number of post-mortem muscle samples from familial and sporadic ALS patients [131]. However, the majority of ALS patients were "pTDP-43-negative" according to their own reference data [132], including 69% of C9-ALS. Interestingly, patients with IBM included in the non-ALS control group were "pTDP-43-positive”. Another study considered semi-quantitative determination of pTDP-43 deposits in cardiac and skeletal muscle (tongue, diaphragm, axial and appendicular) samples from two series of autopsies of patients with post-mortem confirmed ALS with pTDP-43 pathology, compared to other patients with neuromuscular and non-neuromuscular diseases [133]. They found a statistically significant increase of pTDP-43 inclusions in at least one muscle territory in both autopsy series (31.3% in the first, 100% in the second) compared to the neurological controls, especially in skeletal muscle. However, these alterations were unspecific for ALS, as pTDP-43 inclusions were also found in 50% and 42.9% of non-neuromuscular diseases in their respective series.

Concerning the muscular TDP-43 as a potential biomarker, it is quite relevant to point out that the physiological TDP-43 protein and mRNA exert a proven role in muscle regeneration by participating in the assembly of myo-granules [120]. Accordingly, TDP-43 cytoplasmic translocation in patients experiencing muscle regeneration after neurogenic atrophy might be a physiological response instead of a pathological hallmark of TDP-43 proteinopathy. We suggest that the TDP-43 and pTDP-43 deposits in muscle samples from autopsies of ALS patients might inform of a phenomena occurring during late stages of ALS rather than resembling TDP-43 CNS proteinopathy. More extensive research and different methodological approaches, including different primary antibodies and in vivo muscle biopsies, are required to determine whether TDP-43 pathology in muscle biopsy could become a feasible in vivo biomarker for clinical use in ALS and other proteinopathies.

#### Other tissues

Riva et al. performed a novel approach by detecting TDP-43 in motor neuron biopsies from patients with ALS and other neuromuscular diseases by means of immunohistochemical procedures, considering anti-NTD and anti-pTDP-43 antibodies as primary antibodies. They found significantly increased TDP-43 and pTDP-43 both in axons and in Schwann’s cells, and TDP-43 aggregates in ALS and IBM patient motor nerves even without axonal degeneration, pointing out that TDP-43 aggregation is likely an early event in the pathogenesis of motor nerve degeneration [121]. Aggregation of pTDP-43 accumulation was also observed within intramuscular nerve bundles in post-mortem muscle samples from a cohort of ALS patients [134]*.*

No studies were found on TDP-43 deposits in other tissues, such as adipose tissue or liver cells in humans. As muscle and nerve biopsies are more invasive than skin samples, patients might be exposed to higher surgical complications. Further research is required, and ethical issues must be considered to determine whether the diagnostic benefit of analyzing these tissues is worth the risk of the biopsy procedure.

## Upcoming challenges

It is essential to recognize that TDP-43 expression and function change with aging, not only in FTD-ALS patients, but in healthy individuals as well. In human motor cortex tissues, an investigation revealed escalating DNA demethylation with age in the autoregulatory region of *TARDBP* 3’ untranslated region. This process leads to reduced alternative splicing of TDP-43 and a consequent increase in TDP-43 expression [135]. This intriguing finding could elucidate the considerable variability of TDP-43 levels among patients and controls, emphasizing age as a potential confounding factor. Establishing normative values or, at the very least, describing how TDP-43 levels vary with age, appears to be a crucial preliminary step before achieving a reliable biomarker.

Another relevant issue in TDP-43 detection is the methodology used. Most studies are based on Western blotting, immunohistochemistry or immunofluorescence microscopy, with the use of an anti-TDP-43 primary antibody for protein labeling. A few studies detected TDP-43 pathology in cells by means of polyclonal antibodies against the NTD of the protein, finding no differences compared to healthy controls [111, 116, 127]. Only one study [115] found increased cytoplasmic and decreased nuclear TDP-43 in immortalized, monoclonal PBMCs from a small cohort of ALS patients, including one *C9ORF72*-ALS, compared to healthy controls, resembling TDP-43 mislocalisation. On the other hand, studies using antibodies against CTD epitopes did show significant differences between patients and controls. Of note, the CTD antibodies do recognize the truncated CTFs of ~ 35 and ~ 25 kDa, which represent an essential part of the pathologic signature of brain TDP-43 proteinopathy [136]. Therefore, the choice of the primary antibody used seems critical for adequate detection. Reported evidence suggests the use of anti-CTD antibodies instead of anti-NTD, to better detect pathological forms and achieve more consistent results.

Unfortunately, a number of studies lack written information on the primary antibodies used [102, 106, 110, 118, 119, 123]. Although these studies did find increased TDP-43 cytoplasmic deposits in skin and muscle cells from ALS and FOSMN patients compared to controls, their results could not be comparable nor replicable.

Antibodies against pTDP-43 are less commonly used. Two aforementioned studies found an increased amount of pTDP-43 in *GRN*-deficient lymphoblasts [111] and PBMCs from a small number of ALS patients [115]. Rubio et al. discussed that the anti-pTDP-43 might be unnecessary, as anti-CTD antibodies can efficiently detect both native and phosphorylated TDP-43 [122]. In fact, an additional ~ 45 kDa band is often found in TDP-43 immunoblots, corresponding to the post-translational modifications of TDP-43, either phosphorylation and/or ubiquitination [9].

## Conclusions

We support that in vivo classification of TDP-43 proteinopathies is crucial for a comprehensive understanding of the biological mechanisms underlying these neurodegenerative disorders. Additionally, it plays a pivotal role in steering future research focused on developing disease-modifying treatments. To date, the detection of TDP-43 in biofluids has not been very successful. Instead, cellular and tissue-based detection of pathologic species of TDP-43, alone and/or with other clinical, neuroimaging and analytic biomarkers, might be a more promising avenue for its clinical use as a disease biomarker. Current evidence indicates the presence of measurable extra-neural TDP-43 pathology, which seems one of the most promising approaches for evaluating altered TDP-43 in TDP-43 proteinopathies. Further research and efforts are underway to standardize methods to detect cell-based TDP-43 for clinical use across research groups, which is essential for obtaining reliable results.

## Data Availability

Not applicable.

## Abbreviations

AD: Alzheimer’s disease
ADEV: Astrocytes-derived extracellular vesicle
ALS: Amyotrophic lateral sclerosis
APOE: Apolipoprotein E
CNS: Central nervous system
CSF: Cerebrospinal fluid
CTD: C-terminal domain
CTF: C-terminal fragment
DLB: Dementia with Lewy bodies
DTI: Diffusion tensor imaging
EV: Extracellular vesicle
FDG: (^18^F)-fluorodeoxyglucose
FOSMN: Facial onset sensory and motor neuropathy
FTD: Frontotemporal dementia
FTD-TDP: Frontotemporal dementia with TDP-43 proteinopathy
HC: Healthy controls
HD: Huntington's disease
IBM: Inclusion body myositis
LATE-NC: Limbic-predominant age-related TDP-43 encephalopathy neuropathological changes
MRI: Magnetic resonance imaging
NDEV: Neuronal-derived extracellular vesicles
NLS: Nuclear localization sequence
NTD: N-terminal domain
PBMC: Peripheral blood mononuclear cell
PET: Positron emission tomography
p-tau181: Phosphorylated tau 181
pTDP-43: Phosphorylated TDP-43
TDP-43: Transactive response DNA-binding protein 43 kDa
